# KITLG Promotes Glomerular Endothelial Cell Injury in Diabetic Nephropathy by an Autocrine Effect

**DOI:** 10.3390/ijms231911723

**Published:** 2022-10-03

**Authors:** Jiun-Chi Huang, Szu-Chia Chen, Wei-An Chang, Wei-Wen Hung, Ping-Hsun Wu, Ling-Yu Wu, Jer-Ming Chang, Ya-Ling Hsu, Yi-Chun Tsai

**Affiliations:** 1 Department of Internal Medicine, Kaohsiung Municipal Siaogang Hospital, Kaohsiung Medical University, Kaohsiung 812, Taiwan; 2 School of Medicine, College of Medicine, Kaohsiung Medical University, Kaohsiung 807, Taiwan; 3 Division of Nephrology, Kaohsiung Medical University Hospital, Kaohsiung Medical University, Kaohsiung 807, Taiwan; 4 Division of Pulmonary and Critical Care Medicine, Kaohsiung Medical University Hospital, Kaohsiung Medical University, Kaohsiung 807, Taiwan; 5 Division of Endocrinology and Metabolism, Kaohsiung Medical University Hospital, Kaohsiung Medical University, Kaohsiung 807, Taiwan; 6 Graduate Institute of Clinical Medicine, College of Medicine, Kaohsiung Medical University, Kaohsiung 807, Taiwan; 7 Drug Development and Value Creation Research Center, Kaohsiung Medical University, Kaohsiung 807, Taiwan; 8 Graduate Institute of Medicine, College of Medicine, Kaohsiung Medical University, Kaohsiung 807, Taiwan; 9 Division of General Medicine, Kaohsiung Medical University Hospital, Kaohsiung Medical University, Kaohsiung 807, Taiwan; 10 Liquid Biopsy and Cohort Research, Kaohsiung Medical University, Kaohsiung 807, Taiwan

**Keywords:** KITLG, advanced glycation end-products, endothelial–to–mesenchymal transition, glomerular endothelial cell, diabetic nephropathy, biomarker

## Abstract

Diabetic nephropathy (DN) is an increasing threat to human health. The impact of hyperglycemia or its metabolites, advanced glycation end-products (AGEs), on glomerular endothelial cells (GECs) and their pathophysiologic mechanisms are not well explored. Our results reveal that AGEs increased the expression and secretion of the KIT ligand (KITLG) in GECs. Both AGEs and KITLG promoted endothelial-to-mesenchymal transition (EndoMT) in GECs and further increased the permeability of GECs through the AKT/extracellular-signal-regulated kinase pathway. Inhibition of KITLG’s effects by imatinib prevented AGE-medicated EndoMT in GECs, supporting the belief that KITLG is a critical factor for GEC injury. We found higher KITLG levels in the GECs and urine of db/db mice compared with db/m mice, and urinary KITLG levels were positively correlated with the urinary albumin-to-creatinine ratio (ACR). Furthermore, type 2 diabetic patients had higher urinary KITLG levels than normal individuals, as well as urinary KITLG levels that were positively correlated with urinary ACR and negatively correlated with the estimated glomerular filtration rate. KITLG plays a pathogenic role in GEC injury in DN and might act as a biomarker of DN progression.

## 1. Introduction

Approximately 463 million adults have diabetes mellitus (DM) worldwide, and its prevalence has doubled in the past 20 years [1]. DM remains one of the greatest challenges to healthcare systems in the 21st century. In particular, diabetic patients are more susceptible to kidney damage and the subsequent development of chronic kidney disease (CKD). Approximately 30 to 40% of patients with diabetes are estimated to develop CKD through the course of their disease [2,3]. Diabetic nephropathy (DN) is associated with an increased risk of mortality and is the leading cause of CKD and end-stage kidney disease (ESKD) [4,5,6]. Despite advances in treatment of DN [7,8], the complications and economic burden associated with DN are still challenging to patients and healthcare providers. Innovative biomarkers as well as novel treatment strategies for halting the progression of kidney damage in patients with DN are imperative.

Kidney injury caused by DM primarily involves the glomerulus. In the early stage of DN, the histological features include mesangial matrix expansion and glomerular basement membrane thickening [9]. DM-induced changes in dynamic and structural factors lead to glomerular hyperfiltration. Along with the progression of DN, decreased glomerular filtration surface area, nodular sclerosis, advanced glomerulosclerosis, and kidney fibrosis are found [9]. Albuminuria, a declining glomerular filtration rate, and eventually kidney failure comprise the hallmarks of kidney damage [10]. The luminal surface of the glomerular capillaries is covered by glomerular endothelial cells (GECs), which are in direct contact with circulating factors. With the exposure of high blood glucose, GECs are vulnerable to hyperglycemia-induced injury, leading to substantial changes in glomerular permeability, modification of intracellular signaling, and phenotypic switching [11,12]. Furthermore, advanced glycation end-products (AGEs), metabolites of hyperglycemia, increase the production of reactive oxygen species (ROS) and activate the protein kinase C (PKC) pathway, the nuclear factor kappa light chain enhancer of activated B cells (NF-κB), and transforming growth factor-β (TGF-β), and then promote inflammation and kidney fibrosis [13,14]. Changes in fatty acids, lipotoxicity, and inflammation—similar to those seen in metabolic syndrome and CKD—appear to facilitate the progression of DN through the cellular damage of podocytes, as well as mesangial and endothelial cells [15,16]. GEC dysfunction plays a critical role in the pathogenesis and progression of DN [17]. Accumulating evidence suggests that high glucose (HG) stimulates GECs to undergo endothelial-to-mesenchymal transition (EndoMT) [18,19,20,21]. With the hallmarks of the loss of tight junctions and endothelial markers, cytoskeleton reorganization, and the acquisition of the expression of mesenchymal proteins [22], EndoMT seems to act as the key link between GEC dysfunction and renal fibrosis through multiple signaling pathways [23]. Thus, the elucidation of the key pathogenic mechanisms and the search for potential therapeutic targets for blocking the development of EndoMT in DN represent urgent tasks.

The KIT ligand (KITLG), also named the stem cell factor, is expressed in human endothelial cells [24]. KITLG has various biological functions, including the regulation of proliferation, migration, and apoptosis of endothelial cells [25,26,27]. The level of KITLG is enhanced in the kidneys of diabetic ApoE^−/−^ mice [28]. A recent study using an RNA sequencing experiment found that the expression of KITLG was upregulated in glomeruli among patients with DN [29], suggesting that KITLG may participate in the development of DN. However, the role of KITLG in GECs still remains unexplored. Therefore, the aim of this study is to investigate the impact of KITLG and its signaling pathway in GEC injury of DN.

## 2. Results

### 2.1. AGEs Increased Vascular Permeability of GECs

**Figure 1:**
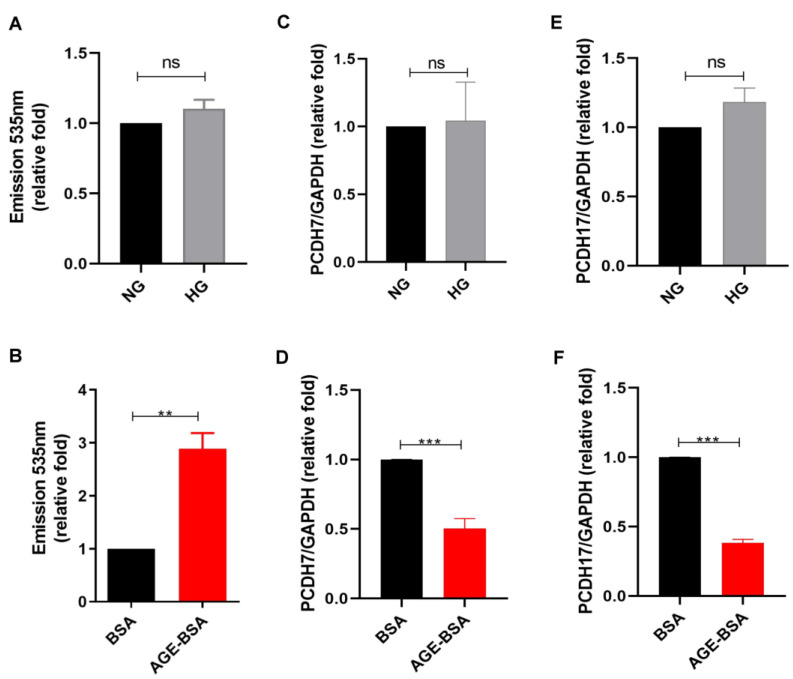
AGEs increased vascular permeability of GECs. (**A**,**B**) The permeability of HGECs was examined after treatment with NG (5.5 mM), HG (25 mM), BSA (300 μg/ml), or AGE–BSA (300 μg/ml) for 48 h (*n* = 3) using fluorescein isothiocyanate (FITC)–dextran dye. (**C**–**F**) PCDH7 and PCDH17 mRNA were assessed in HGECs treated with NG, HG, BSA, or AGE–BSA for 24 h (*n* = 3). ns = not significant, ** *p* < 0.01, *** *p* < 0.001 by Student’s *t*-test.

To investigate the influence of HG on the permeability of GECs, we assessed the effect of HG and its metabolites, AGEs, on the leakage of GECs. A transendothelial permeability assay was used to examine the permeability of human GECs (HGECs) treated with normal glucose (NG), HG, bovine serum albumin (BSA), or AGE–BSA, and we found that AGEs, not HG, increased the vascular permeability of HGECs (Figure 1A,B). According to our previous study, AGEs decreased cell tight junction protein expression, such as that of protocadherin 7 (PCDH7) and protocadherin 17 (PCDH17), in coronary artery endothelial cells [30]. Thus, we examined the effect of HG or AGEs on HGECs and found suppressed mRNA levels of PCDH7 and PCDH17 in HGECs treated with AGEs, which is consistent with the vascular permeability results. HG did not affect PCDH7 and PCDH17, while AGEs decreased their levels in HGECs. These results indicate that AGEs play a role in glomerular endothelium injury within the microenvironment of DN.

### 2.2. AGEs Increased KITLG Expression in GECs

**Figure 2:**
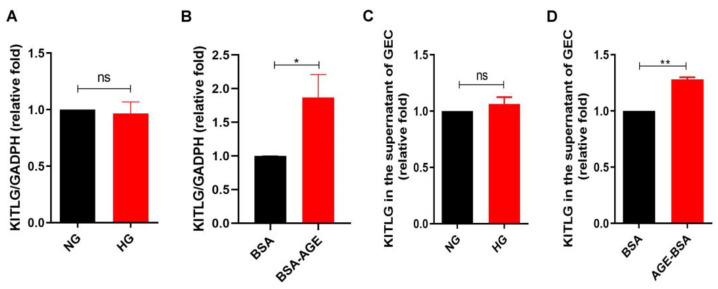
AGEs increased KITLG expression in GECs. (**A**) KITLG mRNA expression in HGECs treated with NG (5.5 mM) or HG (25 mM) for 24 h (*n* = 3). (**B**) KITLG mRNA expression in HGECs treated with BSA (300 μg/ml) or AGE–BSA (300 μg/ml) for 24 h (*n* = 3). KITLG mRNA levels were assessed by quantitative real-time polymerase chain reaction (qRT-PCR). (**C**,**D**) KITLG protein level in the supernatant of HGECs treated with NG, HG, BSA, and AGE–BSA for 48 h. The level of KITLG was determined by an ELISA kit. The bar graph represents the mean ± S.E.M. ns = not significant, * *p* < 0.05, ** *p* < 0.01 by Student’s *t* test.

Since KITLG may play a potential role in DN [28,29], we investigated whether HG or AGEs affect KITLG expression in GECs. HG did not affect KITLG expression at the mRNA level in HGECs (Figure 2A). Conversely, the KITLG mRNA level was increased in HGECs treated with AGE–BSA for 24 h (Figure 2B). We further examined KITLG at the protein level in a supernatant of HGECs treated with NG, HG, BSA, and AGE–BSA for 48 h using enzyme-linked immunosorbent assay (ELISA), and we found that AGE–BSA, not HG, increased KITLG levels in a supernatant of HGECs (Figure 2C,D). These findings suppose that AGEs, but not HG, enhanced the expression and secretion of KITLG in GECs.

### 2.3. KITLG Promoted EndoMT and Furthered the Increase in Permeability of HGECs through AKT (Protein Kinase B)/Extracellular-Signal-Regulated Kinase (ERK) 1/2 Signaling Pathway

**Figure 3:**
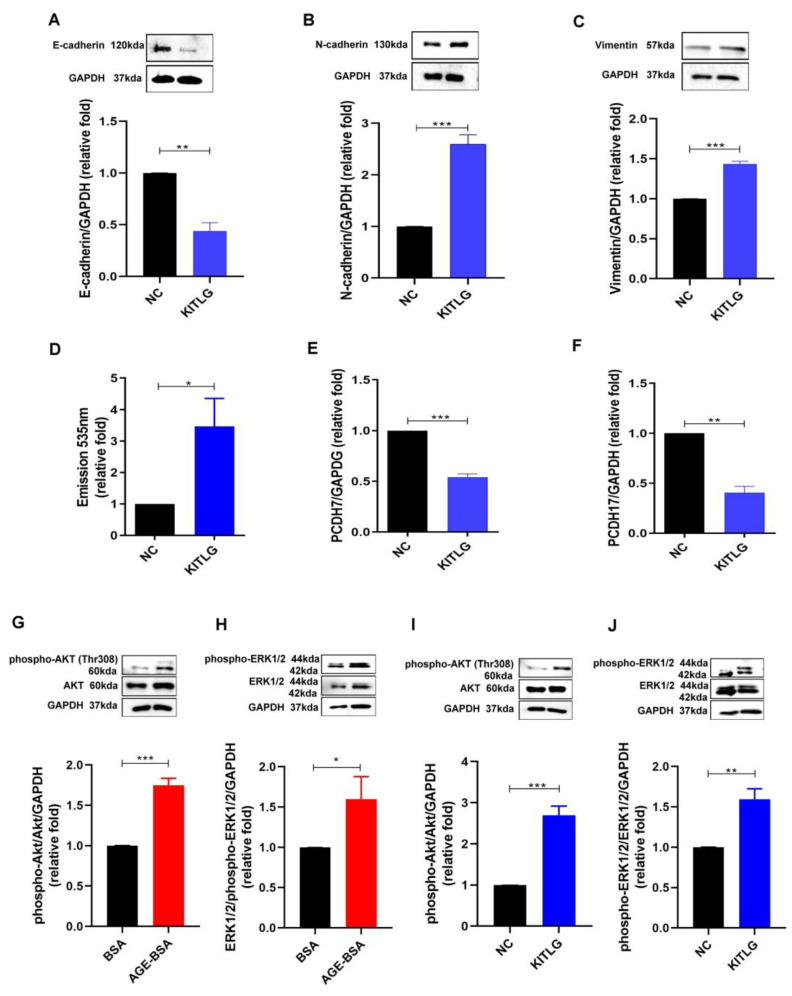
KITLG promoted EndoMT and furthered the increase in permeability of GECs through the AKT/ERK1/2 signaling pathway. EndoMT markers, including E-cadherin, N-cadherin, and vimentin levels, were assessed in HGECs treated with BSA (300 μg/ml) or AGE–BSA (300 μg/ml) for 48 h (*n* = 3). (**A**–**C**) After treatment with KITLG (10 ng/ml) for 48 h, EndoMT was examined in HGECs using Western blotting (*n* = 3). (**D**) The permeability of HGECs was examined after treatment with normal control (NC) and KITLG (10 ng/ml) for 48 h using FITC–dextran dye (*n* = 3). (**E**,**F**) PCDH7 and PCDH17 mRNA were assessed in HGECs treated with NC and KITLG for 24 h (*n* = 3). (**G**–**J**) The level of phospho-AKT/AKT and phospho-ERK1/2/ERK1/2 protein expression in HGECs treated with BSA, AGE–BSA, NC or KITLG for 6 h were examined using Western blotting. * *p* < 0.05, ** *p* < 0.01, *** *p* < 0.001 by Student’s *t*-test.

EndoMT has been indicated as one of the major pathophysiologic mechanisms of DN [22]; thus, we investigated whether KITLG promoted EndoMT in HGECs. KITLG decreased the expression of E-cadherin and increased the expression of mesenchymal markers such as N-cadherin and vimentin, promoting the EndoMT process in HGECs (Figure 3A–C). In addition, KITLG also enhanced the permeability of HGECs (Figure 3D) and suppressed the expression of tight junction PCDH7 and PCDH17 at the mRNA level (Figure 3E,F). Furthermore, we examined the KITLG-mediated signaling pathway in AGE-induced glomerular endothelium injury in DN, and found that both AGEs and KITLG increased the phosphorylation of AKT and ERK1/2, meaning that KITLG may contribute to EndoMT and may further impair the endothelial permeability induced by AGEs in HGECs through the AKT/ERK1/2 pathway.

### 2.4. Imatinib, as a KITLG Inhibitor, Rescued the Glomerular Endothelial Injury Induced by AGEs

**Figure 4:**
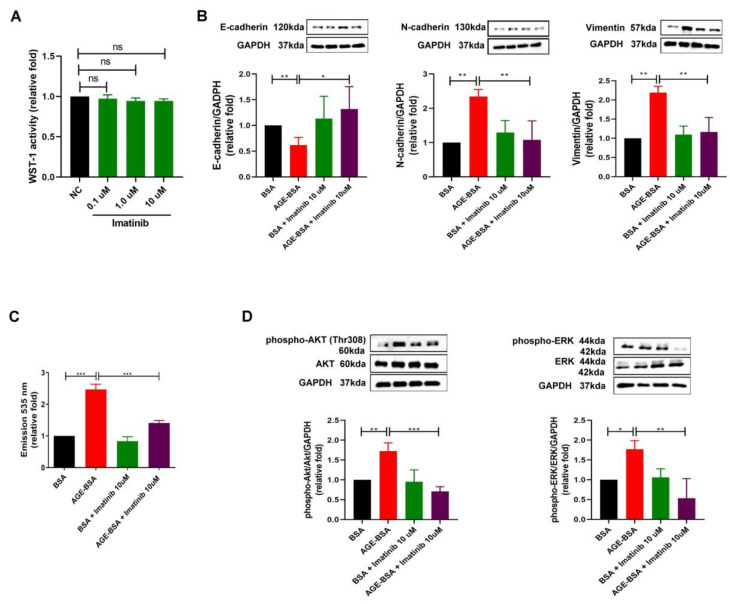
Imatinib rescued the glomerular endothelial injury induced by AGEs in HGECs. (**A**) The viability of HGECs treated with normal control (NC) and different concentrations (0.1 μM, 1.0 μM, and 10 μM) of imatinib was measured using WST-1 assay at 48 h (*n* = 3). (**B**) After pre-treating NC or imatinib (10 μM) for 1 h, EndoMT markers, including E-cadherin, N-cadherin, and vimentin protein levels, were assessed in HGECs treated with BSA (300 μg/mL) or AGE–BSA (300 μg/mL) for 48 h (*n* = 3). (**C**) After pre-treating NC or imatinib (10 μM) for 1 h, the permeability of HGECs was examined after treatment with BSA or AGE–BSA for 48 h using FITC–dextran dye (*n* = 3). (**D**) Phospho-AKT/AKT and phospho-ERK1/2/ERK1/2 expression after NC or imatinib pre-treatment in HGECs treated with BSA or AGE–BSA for 6 h (*n* = 3). Protein levels were examined using Western blotting. ns = not significant, * *p* < 0.05, ** *p* < 0.01, *** *p* < 0.001 by one-way analysis of variance (ANOVA) followed by a post hoc test adjusted with a Tukey correction.

According to the positive role of KITLG in AGE-induced glomerular endothelial injury in DN, we used imatinib, as a KITLG inhibitor, to further examine whether the suppression of KITLG rescued the glomerular endothelial injury induced by AGEs. Firstly, imatinib did not change the viability of HGECs (Figure 4A), indicating that imatinib did not possess cytotoxicity against HGECs. As expected, imatinib reversed the EndoMT process in HGECs induced by AGEs, including the decrease in E-cadherin and the increase in N-cadherin and vimentin (Figure 4B). In addition, imatinib prevented the increase in the permeability of HGECs induced by AGEs (Figure 4C) and suppressed the phosphorylation of AKT and ERK1/2 activated by AGEs in HGECs (Figure 4D). These findings support the pathophysiologic effect of KITLG and the potential therapeutic impact of imatinib on glomerular endothelial injury in DN.

### 2.5. Positive Relationship between KITLG Expression and Kidney Dysfunction in Mice with DN

**Figure 5:**
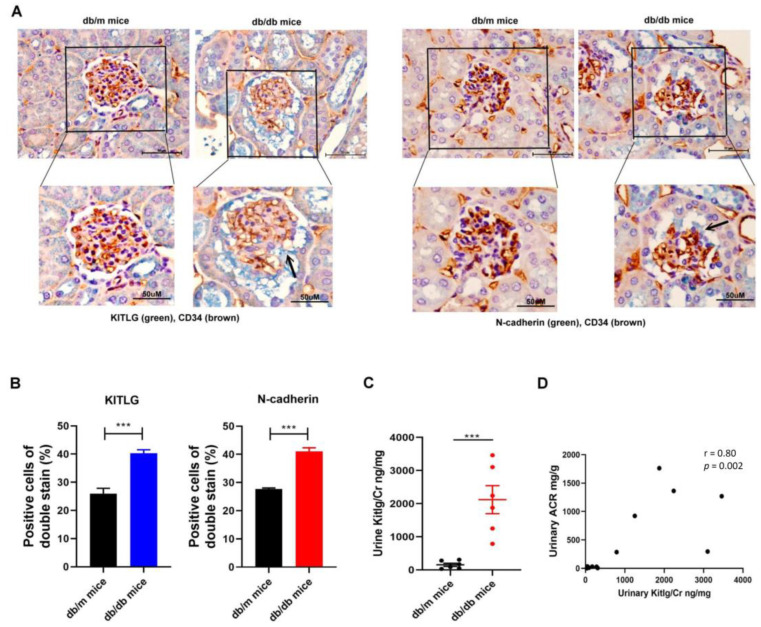
Positive relationship between KITLG expression and kidney dysfunction in mice with DN. (**A**,**B**) The expression of KITLG and N-cadherin in GECs of kidneys in mice is shown. The kidney sections of non-diabetic db/m mice and diabetic db/db mice were co-stained with KITLG (green) and CD34 (brown), as the marker of GECs. (**C**) Urinary KITLG levels were measured in db/m mice (*n* = 6) and db/db mice (*n* = 6) using ELISA. (**D**) The correlation between urinary KITLG levels and urinary albumin/creatinine ratio (ACR). Urine albumin was measured using an immunoturbidimetric assay, and urine creatinine was determined by an enzymatic method. The bar graph represents the mean ± S.E.M. of at least three independent experiments. *** *p* < 0.001 by Student’s *t*-test; the *p*-value of the correlation was assessed by Spearman analysis.

Since KITLG contributed to glomerular endothelial injury in vitro model of DN, we next examined the expression of KITLG in vivo model of DN. Immunohistochemistry (IHC) staining revealed that the expression of KITLG and N-cadherin at the protein level were increased in the GECs of kidney sections of diabetic db/db mice compared with those of non-diabetic db/m mice (Figure 5A,B). We further measured the KITLG levels in the urine of db/m mice (n = 6) and db/db mice (n = 6), and found that db/db mice had higher urinary KITLG levels compared with db/m mice (Figure 5C), and urinary KITLG levels were positively correlated with the urinary albumin-to-creatinine ratio (ACR) in mice (Figure 5D).

### 2.6. Positive Relationship between Urinary KITLG Levels and Kidney Dysfunction in Humans

**Figure 6:**
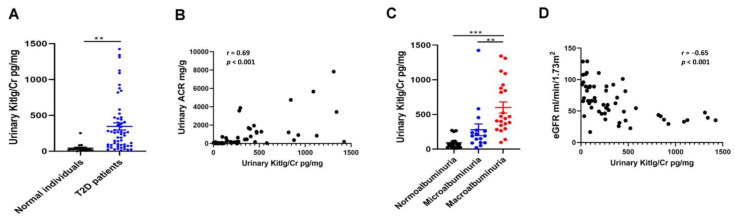
Positive relationship between urinary KITLG levels and kidney dysfunction in humans. (**A**) Urinary KITLG levels were measured in healthy individuals (*n* = 16) and type 2 DM patients (*n* = 56). (**B**) The correlation between urinary KITLG levels and urinary ACR in human participants. (**C**) Urinary KITLG levels among human participants stratified by the severity of urinary ACR. (**D**) The association between urinary KITLG levels and eGFR in human participants. Urine albumin was measured using an immunoturbidimetric assay, and urine creatinine (Cr) was determined by an enzymatic method. Serum Cr was measured by the compensated Jaffé (kinetic alkaline picrate) method. eGFR was calculated using the equation: eGFR = 186 × Serum Cr^−1.154^ × Age^−0.203^ × 0.742 (if female). The bar graph represents the mean ± S.E.M. ** *p* < 0.01, *** *p* < 0.001 by Student’s *t*-test or ANOVA followed by a post hoc test adjusted with a Tukey correction, and the *p*-value of the correlation was assessed by Spearman analysis.

We enrolled 16 normal individuals and 56 type 2 DM patients in our study (Table 1) and measured the KITLG levels in their urine. Type 2 DM patients had higher urinary KITLG levels when compared with normal individuals (Figure 6A). Urinary KITLG levels were positively correlated with urinary ACR (Figure 6B). All study participants with macroalbuminuria had higher urinary KITLG levels than those with normoalbuminuria and microalbuminuria (Figure 6C). More importantly, we found that high urinary KITLG levels were significantly associated with a lower estimated glomerular filtration rate (eGFR) at baseline (Figure 6D). The above results indicate that urinary KITLG has the potential to be a clinical biomarker of kidney injury in type 2 DM patients.

## 3. Discussion

**Figure 7:**
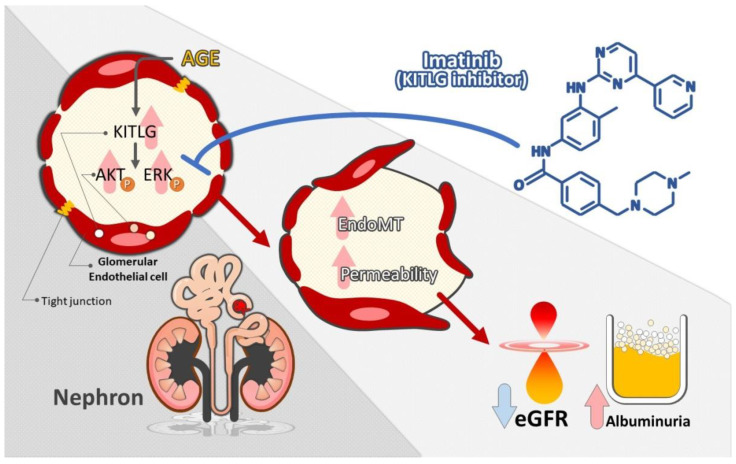
Illustration of the mechanism by which AGEs induce EndoMT and the increase in the permeability of GECs through the KITLG/AKT/ERK1/2 pathway and urinary KITLG as a biomarker of kidney injury in type 2 DM. Furthermore, imatinib reversed AGE-induced EndoMT and impaired the permeability of GECs.

Our study investigates the signal pathway and novel biomarkers of glomerular endothelium injury in the DN microenvironment. Instead of HG, AGEs increased the expression and secretion of KITLG in HGECs, and KITLG promoted EndoMT in HGECs and further increased the permeability of HGECs through AKT/ERK1/2 activation. Conversely, suppression of KITLG’s effects by imatinib ameliorated AGE-mediated EndoMT and increased the permeability of HGECs. Moreover, elevated levels of urinary KITLG/Cr were noted in vivo model of DN and type 2 DM patients compared with normal groups. Urinary KITLG/Cr levels were positively correlated with the severity of albuminuria as a glomerular injury marker. These findings reveal that KITLG could be an indicator of injury in the glomerular endothelium of DN (Figure 7). This study provides new insights into our understanding of the unique mechanisms of KITLG in order to explain the development of DN and as potential biomarkers in clinical patients with type 2 DM.

DN is characterized by abnormalities in the function and structure of glomeruli. Glomerular endothelial injury and dysfunction play a central role in the development and progression of DN [12]. Previous studies revealed the enhanced expression of KITLG in diabetic kidneys; however, the pathophysiologic role of KITLG in DN is unclear [28,29]. KITLG binds to its receptor, c-KIT, on the cell membrane, leading to the activation of two major downstream signal pathways, Ras-PI_3_K-AKT and Ras-MAPK-ERK [25]. These pathways are involved in the regulation of cell survival, differentiation, and proliferation [24]. Aberrant expression of KITLG by autocrine/paracrine stimulation mechanisms has been discovered in various cancers, such as colorectal cancer [31], non-small cell lung cancer [32], glioblastoma [33], and thymoma [34]. Our findings confirm that AGEs enhanced the expression and secretion of KITLG in HGECs, which, in turn, promoted EndoMT and increased the permeability of HGECs by an autocrine effect. In addition to the pathologic role of KITLG, we further found that urinary KITLG levels were positively correlated with albuminuria and negatively correlated with eGFR level in patients with type 2 DM, suggesting that urinary KITLG levels could be a potential clinical biomarker for DN.

One of our novel findings showed that imatinib significantly mitigated AGE-induced EndoMT and further impaired the permeability of HGECs by suppressing KITLG-mediated phosphorylation of AKT and ERK1/2. Imatinib, a tyrosine kinase inhibitor, is used for the treatment of chronic myeloid leukemia and gastrointestinal stromal tumors. Interestingly, previous studies have indicated that imatinib can regress type 2 DM [35] and attenuate DM-associated atherosclerosis [36]. Imatinib may improve insulin sensitivity through the inhibition of the phosphorylation of protein kinases such as AKT, ERK1/2, platelet-derived growth factor (PDGF) and peroxisome proliferator-activated receptor-γ, which are involved in impaired insulin secretion [37]. In addition, imatinib has been reported to relieve EndoMT and pulmonary artery remodeling, as well as preventing the development of pulmonary hypertension [38], suggesting that imatinib might be a potential therapeutic agent for vascular disorders caused by EndoMT. Recently, a phase 2 trial indicated that 26 weeks of treatment with imatinib slowed a decline in β-cell function in people with newly diagnosed type 1 DM [39]. In a mouse model, imatinib effectively ameliorated retinal neovascularization [40]. Taken together, imatinib might represent one of the hopeful therapeutic approaches for different types of DM and their complications. However, whether imatinib affects the pathogenic changes in the microenvironment in DN, especially in the glomerular endothelium, is unknown. One study found that imatinib reversed glomerular and tubulointerstitial injury through the suppression of the PDGF-mediated pathway in diabetic apolipoprotein E-knockout mice [41]. Our results indicate that imatinib exerted a protective effect on the glomerular endothelium through the prevention of EndoMT and further impaired the permeability caused by glucose metabolite AGEs, meaning that imatinib might represent a potential treatment strategy for DN.

Sodium–glucose cotransporter 2 (SGLT2) inhibitors exert a renoprotective effect by halting the progression of DN [42]. Moreover, accumulating evidence suggests that SGLT2 inhibitors attenuate oxidative stress and inflammation in vivo and in vitro models of DN in several ways [43]. Although SGLT2 expression was not found in GECs, empagliflozin protected GECs by limiting podocyte-secreted vascular endothelial growth factor A in DN mice [44]. Further experiments to explore the function and molecular pathways of GECs specifically affected by SGLT2 inhibitors are necessary.

## 4. Materials and Methods

### 4.1. Cell Lines and Cell Cultures

HGECs (Sciencell, Carlsbad, CA, USA, Cat 4000) were cultured in Endothelial Cell Medium (ECM, Cat 1001) plus 5% fetal bovine serum (FBS), according to the manufacturer’s suggestions. Cells were treated with NG (5.5 mM), HG (25 mM), BSA (300 μg/ml, Sigma-Aldrich, St. Louis, MO, USA), AGE–BSA (300 μg/ml, Sigma-Aldrich, MA, USA), KITLG (10 ng/ml, Cat 255-SC, R&D systems, Minneapolis, MN, USA) or imatinib (10 μM, Novartis, Cambridge, MA, USA) for the indicated times.

### 4.2. Permeability Analysis of HGECs

The transendothelial permeability assay was performed using an In Vitro Vascular Permeability Assay kit (EMD Millipore, Burlington, MA, USA). HGECs grown to confluence on collagen-coated inserts were exposed to NG, HG, BSA, AGE–BSA, NC, or KITLG with or without imatinib for 48 h. After treatment, FITC-labelled dextran was added to the top of the cell monolayer for 20 min, and then FITC–dextran was measured across the HGECs’ monolayer to the bottom wells by relative fluorescence excitation at 485 nm and emission at 530 nm using a fluorescence plate reader.

### 4.3. Real-Time Quantitative Reverse Transcription Polymerase Chain Reaction (qRT-PCR)

Oligo (dT) primer and reverse transcriptase (RT; Takara, Shiga, Japan) were utilized to prepare the cDNA after total RNA extraction of cells. The SYBR Green system of QuantStudio3 (Thermo Fisher, Waltham, MA, USA) was used to analyze quantitative RNA. The PCR reaction was carried out with the following temperature profile: 95 °C for 10 min, followed by 40 cycles at 95 °C for 15 s, and 60 °C for 1 min. The relative expression levels of the mRNA in cells were normalized to interval control glyceraldehyde-3-phosphate dehydrogenase (GAPDH). Relative expression was assessed using the 2^−ΔΔ^Ct method. The primers (KITLG, PCDH7, PCDH17 and GADPH) used are listed in Table S1.

### 4.4. Quantification of KITLG Using ELISA

The supernatants of HGECs treated with NG, HG, BSA, or AGE–BSA for 48 h were collected. All urine samples of humans and mice were aliquoted and stored in a −80 °C freezer. The KITLG levels of the supernatants of GECs and the urine of humans and mice were measured using ELISA (Cat DCK00, Cat MCK00, R&D systems, Minneapolis, MN, USA).

### 4.5. Western Blot Analysis

HGECs were lysed in Radio-Immunoprecipitation Assay (RIPA) lysis buffer (EMD Millipore, Burlington, MA, USA). Equal amounts of protein were subjected to 9% SDS-PAGE for electrophoresis, followed by transfer onto a polyvinylidene difluoride (PVDF) membrane, which was blocked with 5% blocking buffer and sequentially immunoblotted with each primary antibody overnight at 4 °C. The membrane was washed with tris-buffered saline (TBS)/Tween-20 (0.2%) and then incubated with a horseradish peroxidase (HRP)-conjugated secondary antibody. The corresponding bands were detected using a chemiluminescent HRP substrate kit (EMD Millipore, Burlington, MA, USA). The chemiluminescence signal was captured using Proteinsimple + Fluorchem Q (Alpha Innotech, San Leandro, CA, USA). Antibodies against N-cadherin (Cat 610921, 1:2000), vimentin (Cat 550513, 1:2000), and E-cadherin (Cat 610182, 1:2000) were obtained from BD Biosciences (Franklin Lakes, NJ, USA) and those against GAPDH (Cat mab374, 1:3000) were obtained from EMD Millipore (Burlington, MA, USA). Antibodies against phospho-AKT (Cat 13038s, 1:1000), AKT (Cat 4691s, 1:1000), phospho-extracellular-signal-regulated kinase1/2 (phosphor-ERK1/2) (Cat 4370s, 1:1000), and ERK1/2 (Cat 4695s, 1:1000) were obtained from Cell Signaling technology (Danvers, MA, USA). The densitometry of the bands was analyzed using Image J software (v1.50d, National Institutes of Health, Bethesda, MD, USA) (https://imagej.net/WelcomeUSA accessed on 15 January 2022).

### 4.6. Experimental Animals

Five-week-old, pathogen-free male db/m mice (non-diabetic animal model), and db/db mice (type 2 diabetic animal model) were obtained from the National Laboratory Animal Center in Taiwan. Urine and kidney samples were collected at the 12th week of age. The kidneys were fixed in 4% paraformaldehyde for IHC staining. All animal experiments in this study were carried out in accordance with the National Research Council’s Guide for the Care and Use of Laboratory Animals and were approved by Kaohsiung Medical University and Use Committee.

### 4.7. IHC Stain

The kidney tissue sections were fixed in 4% paraformaldehyde for IHC. KITLG antibody (1:50, Cat A5672, ABclonal, Woburn, MA, USA) and CD34 antibody, as glomerular endothelial cell markers (1:100, Cat ab81289, Abcam, Cambridge, UK), were used in IHC. The densitometry of KITLG was analyzed using Image J software (v1.50d, National Institutes of Health, Bethesda, MD, USA) (https://imagej.net/WelcomeUSA accessed on 15 January 2022).

### 4.8. Human Study Participants

Fifty-six type 2 DM patients with eGFR ≥30 mL/min/1.73 m^2^ and 16 healthy volunteers were invited to participate. DM was defined as a medical history of DM or the use of anti-diabetes agents. Demographic and medical data were obtained from medical records and interviews with study participants. Serum Cr was measured using the compensated Jaffé method in a Roche/Integra 400 Analyzer (Roche Diagnostics, Mannheim, Germany). eGFR was calculated using the equation of the 4-variable Modification of Diet in Renal Disease Study [45]. Concentrations of serum urea nitrogen (UN) were examined using the enzymatic method (Roche Diagnostics, Mannheim, Germany). This study was approved by the Institutional Review Board of Kaohsiung Medical University Hospital. All participants provided written informed consent in accordance with the Declaration of Helsinki.

### 4.9. Measurement of Urinary ACR Levels

Levels of urinary albumin, as a marker of glomerular injury [46], were assessed using the immunoturbidimetric assay with Tina-quant Albumin Gen.2 (ALBT2, Roche Diagnostics, Indianapolis, IN, USA). Concentrations of urinary Cr were examined using the enzymatic method (Roche Diagnostics, Mannheim, Germany). Concentrations of urinary albumin were corrected by urine Cr before statistical analysis.

### 4.10. Statistical Analysis

The continuous variables were expressed as the mean ± S.E.M or median (25th, 75th percentile), as appropriate. The categorical variables were expressed as percentages. Differences in the distribution of categorical variables were tested using the chi-square test. The significance of differences in continuous variables between the groups was tested using Student’s *t*-test or ANOVA, followed by a post hoc test adjusted with a Tukey correction, as appropriate. The associations among continuous variables were examined by Spearman correlation. Statistical analyses were conducted using SPSS version 22.0 for Windows (SPSS Inc., Chicago, IL, USA) and Graph Pad Prism 9.2.0 (GraphPad Software Inc., San Diego, CA, USA). Statistical significance was set at a two-sided *p*-value of <0.05.

## 5. Conclusions

This study demonstrates the role of KITLG in HGECs within a DN microenvironment, which results in EndoMT in HGECs and enhances the permeability of HGECs. KITLG could be a clinical biomarker of kidney injury in type 2 DM patients, and might represent a potential therapeutic target for DN.

## Figures and Tables

**Table 1:** The clinical characteristics of human participants.

	Normal Individuals n = 16	Type 2 Diabetes n = 56	*p*-Value
Age, years	61.8 ± 5.1	62.7 ± 12.4	0.77
Sex (male), %	65.5	50.0	0.37
Fasting blood glucose, mg/dL	98.3 ± 9.2	131.5 ± 32.5	0.001
Blood urea nitrogen, mg/dL	15.7 (12.7–17.7)	17.5 (13.4–22.7)	0.12
Serum creatinine, mg/dL	0.8 ± 0.2	1.2 ± 0.6	<0.001
Estimated glomerular filtration rate, mL/min/1.73 m^2^	96.5 ± 19.7	62.0 ± 27.1	<0.001
Urinary ACR, mg/g	10.5 (4.9–66.2)	157.1 (27.9–896.2)	<0.001

Data are expressed as numbers (percentage) for categorical variables and as the median (25th–75th percentiles) for continuous variables, as appropriate.

## Data Availability

The datasets used during the current study are available from the corresponding author upon reasonable request.

## Supplementary Materials

The following supporting information can be downloaded at: https://www.mdpi.com/article/10.3390/ijms231911723/s1.
