# Supplementary material for: KITLG Promotes Glomerular Endothelial Cell Injury in Diabetic Nephropathy by an Autocrine Effect
Source: Int J Mol Sci. 2022 Oct 3;23(19):11723. doi: 10.3390/ijms231911723 (PMC9569900; doi:10.3390/ijms231911723)
Supplement: Supplementary file 1 [file ijms-23-11723-s001.zip › ijms-1902271-supplementary.pdf]

International Journal of
Molecular Sciences

MDPI

Table S1. The primers of KITLG, protocadherin 7 (PCDH7), protocadherin 17 (PCDH17) and GADPH.

KITLG_H	Forward tggatgaccttgtggagtgc<br>Reverse TCAGATGCCACTACAAAGTCCT
PCDH7_H	Forward GACTTCGAGGTGTCGGTGAT<br>Reverse GTGGGCAGCAGGTAAAGTGT
PCDH17_H	Forward GGCTGACAGTGACCAAGACA<br>Reverse TCAGAATGACCAAGCACTCG
GAPDH_H	Forward GAGTCAACGGATTTGGTCGT<br>Reverse TTGATTTTGGAGGGATCTCG
